# Digital Twins for Managing Railway Bridge Maintenance, Resilience, and Climate Change Adaptation

**DOI:** 10.3390/s23010252

**Published:** 2022-12-26

**Authors:** Sakdirat Kaewunruen, Mohannad AbdelHadi, Manwika Kongpuang, Withit Pansuk, Alex M. Remennikov

**Affiliations:** 1 Department of Civil Engineering, School of Engineering, University of Birmingham, Birmingham B15 2TT, UK; 2 Department of Mining and Materials Engineering, Prince of Songkla University, Hat Yai 90110, Songkla, Thailand; 3 Center of Excellence in Innovative Construction Materials, Department of Civil Engineering, Faculty of Engineering, Chulalongkorn University, Bangkok 10330, Thailand; 4 School of Civil, Mining and Environmental Engineering, University of Wollongong, Wollongong, NSW 2522, Australia

**Keywords:** digital twin, railway maintenance, asset management, sustainability, BIM, life cycle, circular economy, materials stock flow, resilience, climate change adaptation

## Abstract

Innovative digital twins (DTs) that allow engineers to visualise, share information, and monitor the condition during operation is necessary to optimise railway construction and maintenance. Building Information Modelling (BIM) is an approach for creating and managing an inventive 3D model simulating digital information that is useful to project management, monitoring and operation of a specific asset during the whole life cycle assessment (LCA). BIM application can help to provide an efficient cost management and time schedule and reduce the project delivery time throughout the whole life cycle of the project. In this study, an innovative DT has been developed using BIM integration through a life cycle analysis. Minnamurra Railway Bridge (MRB), Australia, has been chosen as a real-world use case to demonstrate the extended application of BIM (i.e., the DT) to enhance the operation, maintenance and asset management to improve the sustainability and resilience of the railway bridge. Moreover, the DT has been exploited to determine GHG emissions and cost consumption through the integration of BIM. This study demonstrates the feasibility of DT technology for railway maintenance and resilience optimisation. It also generates a virtual collaboration for co-simulations and co-creation of values across stakeholders participating in construction, operation and maintenance, and enhancing a reduction in costs and GHG emission.

## 1. Introduction

At present, passengers choose the railway transport system as it is safe, reliable and fast [1,2,3]. Currently, railway bridges play a crucial part in the transportation system in several countries worldwide [4,5]. However, railway bridges are complex structural systems by nature and can deteriorate under different circumstances, such as through environmental impacts, inadequate inspection and poor maintenance management [6]. The complexity of the structure of railway bridges in the construction and operation stage becomes challenging to manage due to many tasks, activities and components associated with the project. In addition, the traditional construction method (the 2D drafting method) in the management process is not smooth, which leads to loss of information throughout the whole life cycle of the project, resulting in project delays and cost overruns [7]. Building Information Modelling (BIM) can be defined as the 3D digital representation of the physical and functional characteristics of a facility [8]. BIM consist of tools, technologies and interaction policies that are useful to project management, monitoring and operation during the whole life cycle assessment (LCA) process in real-time [9]. In 2016, the UK government announced that collaborative 3D BIM technology is mandatory on all public sector projects, which raised interest within the UK and elsewhere [10]. The most significant advantage of BIM to public sector projects is its ability to share data among stakeholders [11]. Previous studies [12] showed that in the maintenance phase, the energy consumption and the cost is significantly higher than at the other stages throughout the whole life cycle of projects. Moreover, infrastructure renewal, maintenance activities and construction can cause 20% of carbon emissions throughout the railway system, depending on climate change, environmental conditions and the type of rail infrastructure [13]. Therefore, the adoption of BIM in the maintenance and operation stages is needed. BIM can help to reduce the project delivery time, decrease project costs and increase the project quality [14]. Furthermore, the implementation of BIM will reduce the cost of the construction of projects by 20%, as per the UK government construction strategy [15].

**Figure 1:**
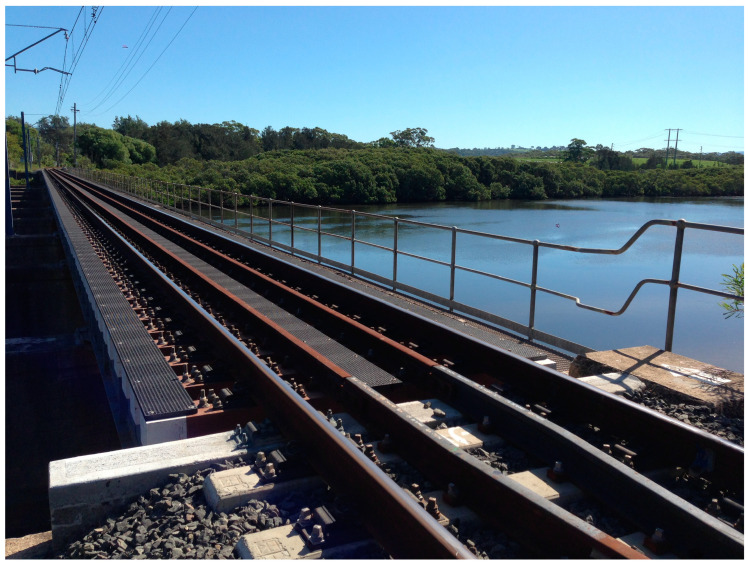
The Minnamurra Railway Bridge in New South Wales, Australia.

Today, LCA is a widely used technique to assess the environmental impact across the life cycle of a product, process or service. When considering an infrastructure’s LCA, it is essential to measure the carbon footprint and consider other environmental effects [16]. In practice, natural hazards, handover defects and environmental conditions, etc., can affect the maintainability of railway infrastructure systems after the construction stage, especially in railway systems, as any deterioration of the risk management would significantly increase the probability of accidents and casualties [17,18,19]. The Minnamurra Railway Bridge (MRB), Australia, has been chosen as a case study to demonstrate the extended application of BIM for the LCA of railway bridges. Several data layers (with the ability of immediate real-time updating) have been embedded into BIM to develop the digital twins (DTs) of the railway bridge. Digital twins can be defined as the virtual representation of physical assets and processes [20]. In railway operation and maintenance, the DT is considered as an integrated rail asset management tool. Unpredicted climate change and improper maintenance management has accelerated the deterioration of the MRB ends which tends to cause more frequent maintenance operations, which is time consuming and costly [21,22]. This paper primarily focuses on assessing the MRB using digital twins to enhance its sustainability throughout its entire life cycle. The digital twin will help in safety management for construction, operational stages and maintenance of the rail bridge. The principal goal of this study is to assess the MRB infrastructure using DTs to enhance the sustainability, resilience and maintenance throughout the whole life cycle of the project to avoid potential risks during the operational stage. The main target is to demonstrate the new applications of digital twins to maintenance and resilience management with an authentic case. As such, a case study using the full-scale Minnamurra Railway Bridge (MRB) in Australia, as shown in Figure 1, has been used to demonstrate the practical insights into the implementation of DTs for circular economy management. This bridge has been chosen due to its unique characteristics including: a special transom bridge trackform, a special arrangement at the bridge ends (using geocells), a special zero-toe-loaded fastening to allow free movement of the rails relative to the steel bridge girder. The old timber bridge transoms have been replaced by FFU composite transoms where new maintenance and condition monitoring plans are required. This aspect underpins the need for digital twins of the railway bridge for managing short- and long-term maintenance, as well as for arranging repairs/reconstructions when it is exposed to extreme conditions.

## 2. Research Background

A critical literature review has been conducted to assess the MRB infrastructure using the digital twin to enhance the sustainability of the construction, and to avoid possible accidents that can occur during operational phases. The literature review establishes the definitions of BIM and LCA, assesses the maintenance of the railway using BIM and proceeds to identifying the problem statement. 

### 2.1. Building Information Modelling (BIM)

BIM is an approach for creating and managing an inventive 3D model simulating digital information that is useful for project management, monitoring and operation of a specific asset during the whole LCA [23]. BIM is also a coordinated digital dataset where relevant parties can access the information on the design and operation and any necessary documents about the project. Any changes within the model are immediately reflected throughout the rest of the project in all views [24]. BIM or the digital twin emphasises the role of all stakeholders in a project and analyses the capabilities of issues in construction, design, operation and maintenance at the early stages, which provides more economical, environmental and time-efficient information than the traditional construction outcomes. From the definition above, it is clear that BIM is not only 3D model software, but its adoption will make dramatic changes in the workflow and project delivery process [25]. According to the National BIM Service, BIM adoption in the UK has been steadily increasing from 39% in 2013 to 48% in 2014, which shows that the architecture, engineering and construction (AEC) industry is moving into implementing BIM services for their future endeavours [26].

### 2.2. The BIM Life Cycle and Maturity Levels

BIM integration spans the entire life cycle of a project. According to Doumbouya et al. (2016), the life cycle stages of a construction project consist of design, production, construction, maintenance and operation. LCA can be defined as the approach to determining a system’s environmental, economic and social impacts. Some previous studies have used LCA on construction, bridges and on structure elements, but its applications are limited in rail projects [16]. Some studies using LCA have been conducted to assess the environmental impacts of the rail sector; a study by Akerman [27], used the LCA approach for a Swedish HSR track, showing a significant benefit in terms of GHG emissions due to the modal shift from road to rail. A case study was chosen by Kaewunruen and Xu [18] to evaluate the effectiveness of the BIM sustainability of the King’s Cross railway station by transforming a 3D model into a 6D building information model where the LCA approach was embedded into the BIM. Using AutoCAD Revit, the model contained a cost- and time- estimation schedule with a carbon emissions calculation and reconditioning assumptions. Furthermore, a study by Kaewunruen and Lian [23] has developed the world’s first 6D BIM (Level 3) for the life cycle management of a railway turnout system using the Revit and Navisworks software. The established digital twin of the railway turnout embraces sustainability, the time estimation schedule and costs throughout the whole life cycle. However, the studies show that BIM applications can produce the added costs of implementation due to the complexity of implementation and the need for staff to learn about BIM.

**Figure 2:**
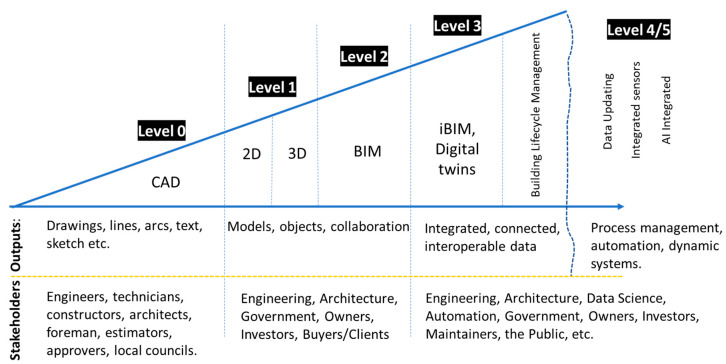
BIM Metrics and maturity levels, modified from [28].

Figure 2 illustrates the ability of several AEC parties to work collaboratively together and exchange information in accordance with the BS Standard 19650-1 [28]. Furthermore, Table 1 shows levels of BIM maturity and the classification at each level, which describes the types of collaborative work, tools and technology used to understand the process at each level. According to Ayinla and Adamu [29], the UK government is operating BIM at maturity Level 2, where management of the full use of collaborative 3D is held in separate BIM disciplines.

### 2.3. Railway Maintenance Using BIM

The railway industry is currently facing the challenge of preventing unpredictable and catastrophic failures, leading to hazardous accidents across the entire network (Dinmohammadi, 2019). Therefore, risk management and maintenance play an essential role in the prevention of accidents such as derailment, degradation and track movement due to the environmental conditions. In common construction projects, the material cost can contribute up to 50% to 60% of the overall cost of the project, and will affect 80% of the project schedule depending on the management method [30]. Moreover, it is complicated to manage and schedule, and to update materials and track component flow throughout the LCA for large projects by adopting traditional construction and asset management methods. A proper maintenance process will provide a safe and comfortable ride and increase the life of the track [31]. Therefore, to enhance green, efficient and sustainable railway systems, effective maintenance and asset management should be considered.

To design an effective railway maintenance schedule, all possible scenarios should be considered. Kaewunruen et al. [16] showed that three essential factors play a crucial role in contributing to the defects of the railway track: (1) the surrounding environmental conditions; (2) unpredictable scenarios that appear during maintenance and operation; and (3) abrasion and erosion causing damage. On the other hand, high accident rates, inexperienced operatives, weak management and inaccurate information on the construction stage occur because the traditional risk assessment is random and lacks up-to-date information. The adoption of BIM can be used as a systematic risk management tool in the development process and it can also be a platform to allow the use of other BIM-related tools for further risk analysis and improve risk identification, as it can perform as a core data generator compared to the traditional risk assessment [32]. Severe climate conditions impact railway operation and safety [33]. A changing climate or temperature could dramatically impact railway infrastructures, such as rail buckling, pavement deterioration and erosion of tracks [34,35].

### 2.4. Resilience in a Railway System

The importance of maintenance is to provide efficiency and productivity of a system by retaining and restoring functions of the technical system throughout the life cycle of the railway infrastructure. Inaccurately performed maintenance activities will lead to deterioration of the track, which will cause significant incidents and accidents [36].

The resilience of a railway transport system can be defined as a comprehensive system that has the capability to produce effective service in normal conditions, as well as to resist and recover quickly from disruptions and disasters [37]. The rapid transport demand and the change in environmental conditions in the urban mobility systems increase congestion in railway networks, which becomes more interdependent and complex to operate, as well as increasing the number of disruptions due to system failure, which increases the difficulty in risk management in all assets [36]. Australia is located between the Pacific Ocean and Indian Ocean in the southern hemisphere [38]. This results in several natural hazards such as floods, heatwaves, droughts and sea-level increases. According to the Australian Institute for Disaster Resilience, the average temperature has increased by 1 °C, and multi-day heatwave events have increased in frequency and duration across many regions of Australia. As the global temperature has risen sharply, it has affected the lifespans of railways and other infrastructures, and the performance of the resilience material used. Moreover, it has had a significant impact on track twisting and buckling and has increased the renewal rate of the materials [36]. A study by Nguyen et al. [39] observed track buckling in Australia during the January 2009 heatwave, demonstrating the dramatic impacts of climate change on railway systems. Therefore, proper maintenance should be considered and monitored in railway systems and infrastructures to avoid such hazards which have implications for the safety of the passengers.

To manage maintenance in practice and to improve the efficiency of maintenance, a powerful simulation in BIM can be applied using the AutoCad Revit software (Suite 2022). Revit is a software for BIM which has tools for creating 3D models and can be used to generate construction documentation [40,41,42]. BIM tools can provide the integration of all inspections of the project and a 3D model visualisation with a vast number of data coordinates being restored.

### 2.5. Railway Infrastructures

A railway transport system is a complex system by nature, and it requires effective maintenance to produce safe, economical and sustainable transportation of goods and passengers [43]. Railway infrastructure can be constructed by a variety of structural assemblies, such as sleepers, rail fasteners, steel rails and foundations. The assemblies consist of ordinary materials, such as timber, steel, rubber and concrete. The most common classical track used in railway infrastructures is the ballasted track, where the rail is placed onto a timber or concrete sleeper. The sleeper sits on a ballast bed (crushed rock), which distributes the loading to the subgrade/formation [44]. The ballast bed can absorb considerable compressive stresses and decrease the effective train load on the ground [45]. The advantages of a ballast track are that it is relatively simple to maintain or construct and has low construction costs, which will dramatically benefit railway developments. However, routine maintenance is always required for a ballast track. In this study, the ballast track and rail bridge track of the MRB has been adopted for demonstrating the digital twin for managing maintenance and resilience.

### 2.6. The Problem Statement

The application of BIM has been widely used to support the construction of buildings, and its technology and methods have shown the potential for benefiting the transportation infrastructure industry [8]. Several studies using a BIM analysis in several constructions showed that, in the construction phase, it has the highest cost consumption [46]. However, an extensive review of 6D BIM applications for transportation structures like railway bridges is still lacking. Moreover, this study aims to address this by adopting an extended BIM for the Australian MRB for better maintenance and resilience management throughout the project’s life cycle. Environmental conditions and design parameters play a crucial role and should be monitored. Material costs and time consumption in construction and maintenance activities for the different components of the railway bridge are also essential factors to be embedded in BIM.

## 3. Materials and Methods

**Figure 3:**
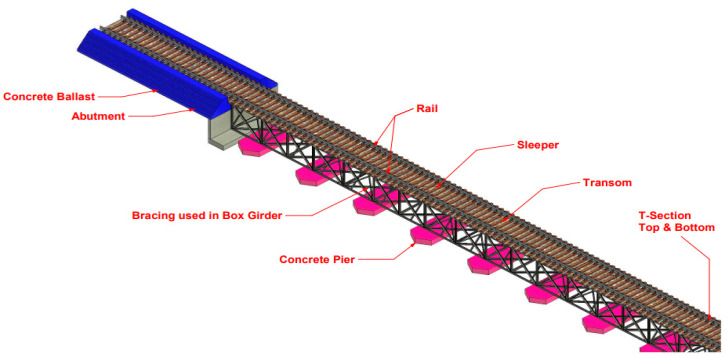
A 3D model of the Minnamurra Railway Bridge developed using AutoCAD Revit v 2022.

**Figure 4:**
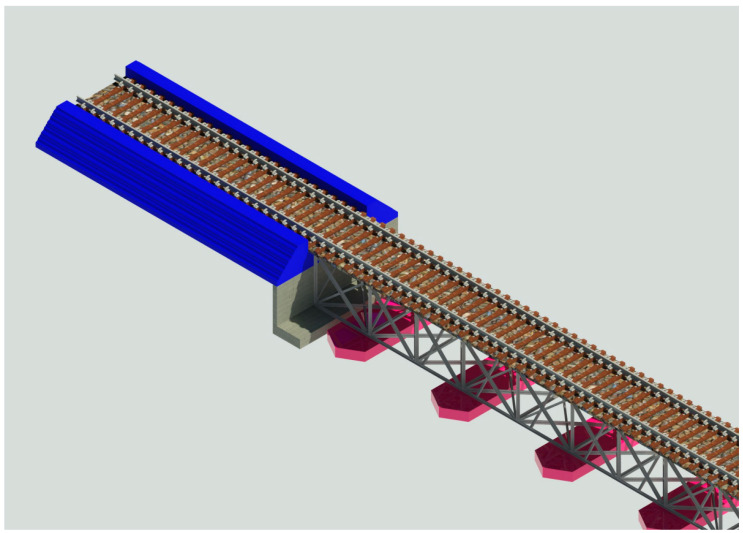
A 3D render view model of Minnamurra Railway Bridge developed using AutoCAD Revit v 2022.

This section provides an approach to the critical methods used within this study to develop digital twins for the MRB to help manage maintenance and resilience by providing a powerful railway bridge section for visualising the workflow in controlled systems. According to the literature review, the whole life cycle can be divided into various levels, showing the different main stages with the critical tasks inside. BIM of the MRB has been modelled using the AutoCad Revit v.2021 software, based on two-dimensions (x and y) or 2D (CAD) accurate drawings obtained from Transport NSW and Sinclair Knight Merz. In contrast, a three-dimensional (x, y and z) or 3D model has been developed using Revit software, as shown in Figure 3. The 3D BIM model will help visualise particular components in detail, as shown in Figure 4 and add coordinated information such as the construction process, materials and costs. The model can then be imported into Navisworks. Navisworks software can combine construction and design data into a single model, with the ability to develop or import schedules from project models and create synchronised project views that combine the Revit and AutoCAD files [40,41] Therefore, the four-dimensional (x, y, z and time) or 4D model can be demonstrated on the timeline of the project, which will enhance the duration of the operation and the maintenance phase of the project. Furthermore, a five-dimensional (x, y, z, time and financial value) 5D version comprises the 4D model and the cost data, which will estimate the project by dividing it into two sections: (1) raw material costs and (2) multi-category costs. The total cost can be created with Revit. A carbon footprint will be analysed using six-dimensions (x, y, z, time, financial and environmental values) or a 6D BIM throughout the life cycle.

### 3.1. Background Information of the Railway Bridge

**Figure 5:**
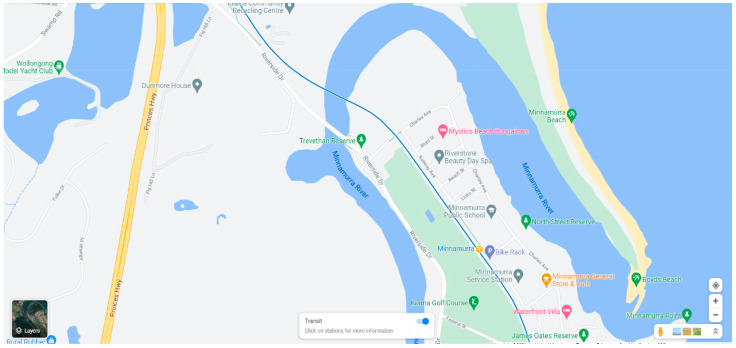
The location of the Minnamurra Railway Bridge (Google Maps).

The Minnamurra Railway Bridge is located in Kiama, New South Wales (NSW), and has been chosen as a case study, as shown in Figure 5. The railway bridge spans the Minnamurra River and is located on the Illawarra Line, which links the south coast of NSW with Sydney [47]. The bridge structure is a steel truss box girder railway, 127.95 m in length and 2 m from the base of the box girder to the top of the rails. In addition, the box girder is supported by concrete piers. Furthermore, the railway bridge consists of 12 spans; spans 2 to 11 are 10.668 m long and span 1 and span 12 are 10.662 m long. Figure 4 shows the components analysed in the 3D model using Revit to provide an adequate maintenance schedule and determine the project’s cost and carbon emissions.

### 3.2. Estimation of Greenhouse Gas Emissions (GHG)

The extent of CO_2_ emissions in the construction industry is very apparent, making it a significant concern for the engineers to have green construction [48]. Moreover, the estimation of GHG is needed throughout the whole LCA of the project. According to the BSI ISO 14064-1:2019 [49], the *GHG emissions* can be calculated using Equation (1):

(1) $$GHG\;emission=\sum \left(f\times I\times \mathrm{GWP}\right)$$

where *f* is the coefficient factor of *GHG emissions*, *I* is number of activities, and GWP is the global warming coefficient. For carbon dioxide, it is 1. Table 2 and Table 3 show the coefficient factors of different materials. Table 2 was extracted from the software Ansys Granta Edupack version 2021 [50], the comprehensive material and process information database. The GHG emissions are mainly related to the materials in the processing and manufacturing stages. By knowing the energy supply and the amount of materials used, carbon emissions can be calculated using the parameter factors (*f*). It is assumed that each track section (e.g., 100 m.) will have similar installation and construction techniques for the track and bridge components so that the components constitute similar raw material ingredients and emit a similar level of carbon footprint [50]. Note that the values in Table 2 and Table 3 are representative indicators and can be adjusted to suit other projects.

### 3.3. Sustainable Maintenance in Railway Systems

The frequency of track inspection is a major aspect that influences the maintainability, reliability and quality of the railway infrastructure, whereas if the regular period of maintenance inspection is taking more time, it will affect the safety of the railway system. Alternatively, if the maintenance inspection period is short, it will significantly increase costs and reduce time efficiency. Moreover, in railway systems the maintenance activities are essential to maintain the structure in a condition that allows it to be used in a safe way [51]. These activities can be divided into two phases: Preventive Maintenance (PM) and Corrective Maintenance (CM). In CM, the failure has occurred in the operation or construction stage and repair activities must be carried out; the damaged item is repaired so that it can achieve its required function [52]. PM is carried out when degradation or deterioration are found with usage and age, and the intention is to reduce the possibility of failure, or repair the aging parts before full damages occur [53]. The MRB is a long-term infrastructure, so a crucial factor is to have adequate maintenance and a proper maintenance schedule to enhance its reliability, safety, sustainability and resistance. PM is considered to be more preferable for the maintainability and sustainability of railway bridges as it reduces costs, it guarantees fewer fault probabilities, the maintenance is performed immediately as required and there are a limited number of monitoring measures [54]. In this study, the LCA will consider a maintenance schedule, cost estimation and GHG estimation in line with the PM approach. 

### 3.4. Frequency of Maintenance and Cost Schedule (5D Model)

Developing a 5D model facilitates the stakeholders or participants of the project to visualise the reconstruction activities and their cost over time. If any design changes occur, the 5D model can update any changes in terms of the budget or schedule. According to Bryde et al. [55], when using the 5D BIM model to explore the impact of changes, all participants can keep the project scope in review, which becomes a trustworthy relationship between designers and owners. Table 4 shows the specific activities of PM in the MRB. The maintenance inspection suggested in the schedule is designed to prevent all degradation or deterioration and to monitor the state of the components and assets. Table 4 demonstrates the material cost depending on the maintenance activity of each component. Moreover, the total maintenance cost consumption can be calculated by multiplying the cost of each material by the number of inspections needed as shown in Equation (2):

(2) Number of Inspection Time×Material cost=maintenance cost consumptio

Life cycle cost can be defined as the cost incurred by a product or service in its life cycle [56]. In this paper the cost of materials per year are calculated for estimating the life cycle cost based on length of the bridge, the material and the components used.

### 3.5. The Material Inventory

The material inventory is essential to the whole project, and effective management which observes the material and components’ flow is necessary. Table 5 shows the components list with its corresponding material in the MRB. Furthermore, common deterioration or damages appearing in the materials, such as steel corrosion, concrete cracks or carbonisation, rail steel wear and rail surface damages, can be accounted for. Throughout the maintenance inspection, the progress of the material checks via the LCA and the damages can be reviewed using BIM. Revit software can be used to observe the material stock so that any requirements can be viewed and checked via the DTs.

## 4. Results

The 6D BIM model comprising the 3D Revit model of MRB, maintenance cost estimation, maintenance timeline schedule and carbon emission estimation was produced using Navisworks and Revit software.

### 4.1. Greenhouse Gas Emissions (GHG)

**Figure 6:**
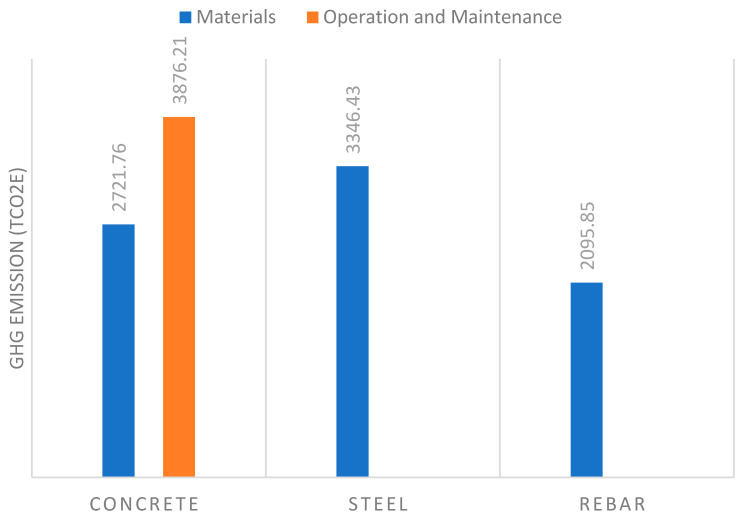
GHG Emissions of materials, operation and maintenance.

The GHG emissions of the materials have been integrated into the manufacturing process. The quantity of the main structural materials of the MRB infrastructure—which are concrete, steel and rebar—can be determined for the calculation of the GHG emissions by using the developed DTs of the MRB infrastructure. Figure 6 shows the level of GHG emissions of the main structural materials and the total carbon emissions during operation and maintenance. The results show that the amount of steel, concrete and rebar are 40.9%, 33.338% and 25.67, respectively. Moreover, the total power consumed is 7,706,182.903 Kw/h per year. The calculation of the total carbon emissions during operation and maintenance, is derived from the power consumed multiplied by the GHG emission factor of the electricity generated, which is 0.503 as provided in Table 2.

### 4.2. The Bill of Quantities

**Figure 7:**
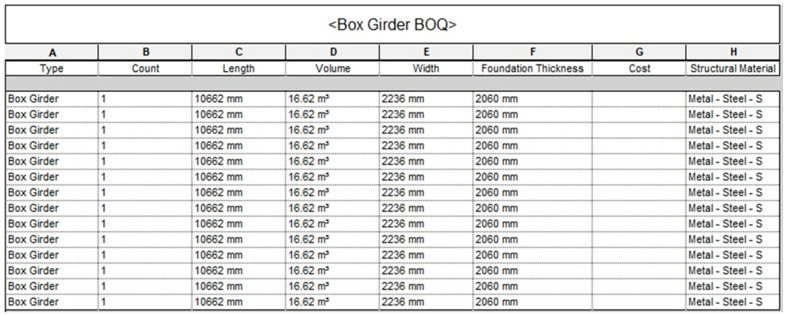
An example of the bill of quantities (BoQ) of steel box girder components installed on the bridge.

The MRB infrastructure consists of a high number of complex components and materials and adopting the traditional inventory method would compromise efficiency. The traditional inventory method in construction projects for producing a bill of quantities, is based on information extracted manually from (2D) drawings and specifications. A traditional bill of quantities is a draft document showing a material plan that outlines part of a project’s management, which typically involves estimating costs and developing a structured framework for construction projects. In addition, the current traditional approach is not smooth, and has several issues such as, the loss of information, the time-consuming preparation and issues of document integration. Nevertheless, Figure 6 shows the bill of quantities for the steel box girder as developed using Revit (i.e., the DT). If any change is made on the Revit model the list will automatically update, which will help to have more time to consider cost alternatives and accurate cost estimates. The DT provides several benefits such as a better visualization and data sharing process, and it emphasises the role of all contractors and stakeholders who are involved in the project. The DT has a significant influence on resilience management in the operation phase and on the maintenance phase. Moreover, in the Minnamurra Railway Bridge system, several elements have affected the maintenance operations in various life cycle phases. Adequate management and task preparations play a crucial role throughout the maintenance operations. For instance, PM can increase the efficiency of costs and time, and decrease project risks, because PM is intended to reduce the possibility of failure or to repair the aging parts in time before full damages occur. To enhance PM activities, the established bill of quantities using Revit can be used to monitor the flow of the material used and the inventory at any time, as shown in Figure 7.

### 4.3. Cost Estimation

The costs have been calculated using Equation (2) and are based on the information provided in Table 4. For example, the cost of track patrolling maintenance inspection is £7.63 every time the inspection is executed; the frequency of this inspection is twice per week so this event will be held 96 times per year, which makes the cost of this inspection £732.48 per year. The total maintenance cost of the railway bridge is about £3098.83; the highest expenditure occurring in the inspection of the railway components, which accounts for 65.72% due to the higher frequency of maintenance in the railway systems.

### 4.4. Maintenance Schedule

**Figure 8:**
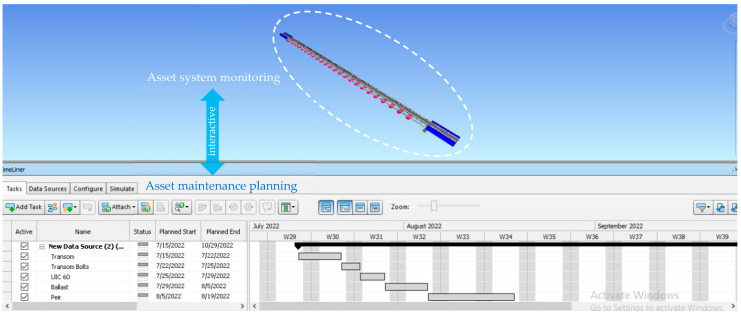
An example of the capability of the BIM-based digital twin to incorporate the timeline maintenance schedule of the Minnamurra Railway Bridge. The BIM-based digital twin can also be further extended to append real-time structural health from live monitoring sensors installed on the bridge.

Navisworks v.2021 is a software that has the ability to link several elements in the Revit model with specific project tasks. Figure 8 illustrates the maintenance schedule information combined with the Revit v.2021 model, which has been developed using Navisworks. After extracting the 3D model information and the bill of quantities from Revit, the model is imported into Navisworks. Navisworks is used in this study to evaluate the integration of the model obtained from Revit and maintenance schedule with the timeline in Navisworks in order to keep the information in BIM current. The material inventory will be updated immediately when any new maintenance activity has been implemented. Moreover, this will help the railway industry to have an effective material management, which can reduce the loss of excessive inventory.

### 4.5. 3D Modelling of the Railway Bridge

The 3D model of the MRB has been developed using Revit v.2021, based on 2D drawings obtained from Transport NSW and Sinclair Knight Merz. The establishment of the 3D model validates the geometry and information inside the corresponding model in order to monitor the accuracy of each detailed part. The Revit model demonstrates the detailed 3D components of a railway bridge, which will help to visualise any hidden complex components. Engineers can use the developed BIM to observe and identify the location or the component that has damages, and whether it requires further maintenance activities. Furthermore, Revit has an enormous database containing all different types of materials where the material type can be chosen and applied to each 3D component. The materials’ information has the component model, dimension, shape and quantity.

## 5. Discussion

The results show that steel has the highest GHG emissions as the majority of the GHG emissions are derived from the box girders. This is due to the majority of the MRB infrastructure components being constructed from steel, and the design of the steel box girder (dimension and volume) being overly large, which contributes to higher material consumption, GHG emissions and costs. A study conducted by Raeisi et al. [57] has developed structural health monitoring (SHM) to reduce the GHG emissions on bridge infrastructures. The study examined the load distribution factor and neutral axis position of two steel box girder bridges to extend the service life of the bridges and the results showed that extending the service life will reduce the use of construction materials, which will decrease the GHG emissions significantly. Generally, steel production is one of the largest total environmental effects of all industrial operations and is one of the major contributors to GHG emissions [58]. Moreover, in 2014 steel accounted for 6.6% of global GHG emissions and 8% of energy related emissions [59,60]. However, several countries have announced strategic visions to achieve net zero GHG emissions by 2050 in a cost-efficient manner, but there is no specific approach which can deliver net zero in terms of steel production and manufacturing, as all approaches lead to significantly high production costs [61].

Currently, there is no specific BIM or DT standard that supports implementation in railway bridges or the bridges industry. The case study of the MRB showed that DTs can be fully integrated with the bridge industry to enhance risks, sustainability and resilience in asset management. The study showed that uncertainties such as environmental impacts will lead to frequent track inspection and the use of DTs can increase the maintenance efficiency when damages occur in the railway bridge components. This is because DTs can: (1) monitor the condition of the bridge such as locating damages accurately; (2) provide real-time information on the infrastructure’s condition through the detecting sensors (3) have the ability to share and visualise data among stakeholders in order to identify technical issues and prioritise maintenance options accordingly; (4) generate virtual collaboration between engineers and stakeholders; (5) identify the capacity of materials and components’ information through the 3D model for maintenance or repair activities; (6) aid engineers in determining life cycle costs and GHG emissions or to develop low cost and low carbon solutions to emerging hazards, as well as improving resilience, sustainability and maintenance of railway bridge infrastructures.

### 5.1. Potential Risk Damages in Materials

In most cases, the materials used to construct the components are the most vulnerable to failure. In practice, over a significant operational life, damages occur in the ageing components and materials [62]. Through the DT database, these issues can be identified and addressed based on the results of local maintenance inspections, repair maintenance records and monitoring sensors. According to Kaewunruen et al. [16], repair techniques and maintenance operations can vary depending on the severity of the failure, and the materials required can differ over the maintenance phase. Moreover, during maintenance inspection several material and components’ failures has been discovered; the following section presents maintenance measures and techniques for repairing damages of the materials.

**Steel/Aluminum Alloy:**

Corrosion is a primary cause of carbon steel bridge corrosion, posing a severe danger to the safety of civil structures and infrastructure systems [63]. Corrosion is considered one of the most common causes of steel bridge deterioration. A steel girder bridge can be affected by several types of corrosion the most common prevalent form is a general loss of surface material, which causes members to thin out over time [64]. The majority of corrosion damage is caused by general corrosion, which is due to electrolyte and oxygen supply. Furthermore, steel corrosion can be caused by chloride ion erosion. The corrosive effects of chloride ions are well known. When they reach the steel bars, a sequence of complex chemical processes take place, damaging the steel bars passive layer and corroding them. Chloride ions impact the steel bars within the concrete and when the concentration reaches a critical level, the steel bar will corrode [65]. Coating the surface of the concrete can prevent future chloride contamination; nevertheless, the steel bars should be replaced if the corrosion is too severe. For steel components such as steel girders with a low corrosion rate, paint coating systems such as polyaniline (PANI) pigmented paint coating can be applied, which is highly corrosion resistant in neutral and acidic conditions and helps to minimise oxidisation [66]. Moreover, wear and rolling-contact fatigue are the two main characteristics that restrict the lifetime of rail tracks; steel rails should be replaced immediately if the wear rate is severe and showing a reduction in cross sectional area [67].

**Concrete:**

Carbonation of concrete occurs due to ingress of atmospheric CO_2_ into concrete, resulting in cracks within the concrete and increasing the steel corrosion rate [68]. Carbonation is a primary cause for excessive cracks in the concrete causing corrosion of bridge steel bars and damage to the bridge structure. Furthermore, three types of concrete surface protection can be used, which are a siloxane pore liner, an epoxy resin coating and an acrylic resin coating to prevent the penetration of aggressive agents such as carbon dioxide [69].

**Railway Bridge Repair Activities:**

Railway bridges are considered the most vulnerable infrastructure and inadequate maintenance, age of the infrastructure, and environmental conditions can have a serious impact on the safety of the structure [12]. Therefore, the replacement, repair and evaluation of bridges are essential in order to reduce the impact of deterioration of the infrastructure components. Moreover, by creating the maintenance activities and entering them into Navisworks and Revit, to develop a maintenance schedule, examples of repair-and-replace maintenance activities can be introduced as shown in Table 6.

### 5.2. Climate and Environmental Impacts

Due to the wide variety of geographical circumstances in Australia, railway networks experience a huge variation of high heat each year. Moreover, the MRB being located in the south coast of NSW, has experienced unpredictable temperatures and weather conditions such as wind, extreme heat, rainfall and snow. The weather conditions and environmental impact implications need to be considered in order to avoid any unanticipated situations that result in infrastructure damage or risk. Climate change is a significant issue to the rail network infrastructures due to the increase in average temperature [70]. Global temperature has risen sharply and it is reported that the mean global temperature will rise by 1.2 °C [71]. Extreme temperature, either extremely hot or cold, can impact the bridge transition areas performance, which consists of rail pads, sleepers, ballast glue and ballast mats. Furthermore, an extremely low temperature decreases the lifespan of rail pads and ballast mats, which will increase the maintenance frequency. On the other hand, high temperatures or heatwaves will cause track buckling and thermal expansion of structural components. In December 2011–2012, Kiama has faced the coolest summer on record with 23.6 °C, and because of the unpredicted weather conditions and the train-imposed loads on the track, the contracted concrete transoms suffered deterioration and an alternative of Fibre Reinforced Foamed Urethane (FFU) transoms has been considered. Moreover, potential environmental impacts such as flooding need to be considered so that further risks are avoided. The MRB is regularly affected by flooding events, as NSW is one of Australia’s most vulnerable states for flood damage, especially within its transportation infrastructure [72]. Table 7 shows the potential damages to the bridge infrastructure due to a flooding event. Therefore, mitigation strategies are essential for infrastructure protection, and effective maintenance will help to decrease the potential failure of the railway [73,74,75,76,77]. Examples of maintenance that could be undertaken are strengthening the soil foundation, installing more drainage systems and replacing the sleepers. This information has been integrated into the digital twins to provide suitable options and integrated decision-making tool for climate change adaptation.

## 6. Conclusions

In practice, the construction of railway bridges is considered a complex and massive project that consists of a large number of engineers, stakeholders and organisations that can benefit from sharing and visualising the data. The traditional asset management has suffered from ineffectiveness, which has led to drastic accidents in construction, operation and maintenance phases. DTs performing as a visual and information platform for project participants through the use of Revit and Navisworks, can improve the efficiency and reduce the risks in the project process. DTs can act not only as an information platform, but they also have the ability of immediate real-time updating and enable access to several data layers throughout the whole life cycle of a project. By using DTs all project parties can collaborate, visualise and share data of the updated progress. DTs can help to improve sustainability, resilience and maintenance by enabling an effective asset management tool; they can evaluate and determine cost estimation, GHG emissions and time schedules. This paper mainly studies the ability of DTs to achieve the project sustainability of a railway bridge infrastructure through the integration of BIM and Navisworks. The Minnamurra Railway Bridge, Australia, has been chosen as a case study to demonstrate the extended application of BIM (i.e., DTs) for the LCA of railway bridges. In addition, the integration of the 3D model using Revit and Navisworks, the maintenance timeline schedules, cost estimations and GHG emissions have been obtained to develop the 6D model. The present case study has also demonstrated that the material inventory can be obtained and monitored using Revit (i.e., BIM). Moreover, the study showed that the maintenance and repair activities should be considered and that Navisworks can help to monitor the activities efficiently. It is noteworthy that climate and environmental factors have a crucial impact on track inspection activities, and the resilience of the materials and components is essential to the MRB infrastructure reliability and safety. Furthermore, improper maintenance management has accelerated the deterioration of the MRB ends, which has tended to cause more frequent maintenance operations, which is time consuming and costly. This study showed that DTs can enhance asset management, operation and maintenance to a more sustainable level for railway infrastructures. This study reveals some limitations of the DT in dealing with the durability aspects of components (e.g., corrosion and ettringites, etc.) where additional co-simulations will be required to gain better insights for effective maintenance management. Future work will incorporate real-time weather conditions into BIM, which can be used to assess bridge vulnerability to natural hazards.

## Figures and Tables

**Table 1:** Level of BIM maturity.

Classification	Description
Level 0 (CAD)	Unmanaged CAD, in 2D, with paper (or electronic paper or blue print) data exchange
Level 1 (CAD, Solids Work)	Managed CAD in 2D or 3D format with a collaborative tool providing a common data environment with a standard approach to data structure and format. Commercial data separate.
Level 2 (BIM)	A managed 3D environment held in separate discipline ‘BIM’ tools with data attached; commercial data will be managed by enterprise resource planning software and integrated by proprietary interfaces or bespoke middleware. The dimension of information can be further extended to 4D to include construction sequencing and 5D to include cost information.
Level 3 (Digital Twins)	A fully integrated and collaborative process enable by ‘web service’ or an interactive network (e.g., intranet, cloud, co-simulation, Navisworks link, etc.) and compliant with emerging industry foundation class standards. Generally, at least 6 dimensions (6D) of information will be integrated. In addition to physical dimensions in 3D (width, length, depth), the dimension of information will traditionally include 4D for construction sequencing, 5D for cost information, and 6D for project life-cycle management information. An additional information layer (i.e., 7D) can also incorporate carbon footprint, environmental impacts and toxicity information.
Level 4 (Digital Twins)	Integration of inspection data (routine condition monitoring) or interactive real-time sensors (sensing for spontaneous actions, transient responses, ambient environments, crowdsensing or live human perceptions) in the BIM/Digital twins.
Level 5 (Digital Twins)	Automation for decision making. A full integration of inspection data, real-time sensing data, and co-simulations for predictions (using constitutive and empirical models, numerical or analytical simulation methods, machine learning and artificial intelligence, data-driven physics informed techniques, etc.).

**Table 2:** GHG Emission factors (f) per one unit of material or by energy type [50].

Material	Country	Unit	CO_2_e (kg CO_2_e)
Concrete	AUS	m^3^	116.45
Steel	AUS	kg	2.25
Cement	AUS	kg	0.437
Rebar	AUS	kg	1.96
Electricity generated	AUS	kwh	0.503
Fuel oil	AUS	l	3.109
Diesel	AUS	l	2.499

**Table 3:** The embodied CO_2_e factors (f) [50].

Component	Country	Unit	CO_2_e (kg CO_2_e)
Ballast	AUS	kg	0.005
Road base/stabilised soil	AUS	kg	0.0051
Insulators	AUS	kg	3
Resilient fastening system	AUS	kg	3

**Table 4:** Heavy rail preventive maintenance and estimated costs in the Minnamurra Railway Bridge. Note that this is an estimation and can be adjusted to suit any future change in activities, frequencies, or costs.

Item	Activity	Frequency	Material Cost (£)
Railway track components (rail, guard rail, transoms, etc. over the bridge and bridge ends)	Integrated track patrolling maintenance inspection	Twice per week	7.63
	Track clearance	Every year	321.41
	Welded track stability analysis inspection	Every year	561.25
	Rail wear and condition inspection	Every year	10.53
	Rail corrosion inspection	Every year	11.36
	Insulated joints inspection	Every 6 months	12.31
	Detailed sleeper and resilient baseplate inspection	Every 2 years	67.41
	Ballast inspection	Every year	326.12
	Abrasion rate and elevation position inspection	Every 6 months	6.76
	Track alignment and rail profile inspection	Every 2 months	83.86
	General structural inspection of transoms and fastening systems	Every year	26.34
Bridge Components and other infrastructures	Minor concrete patching and repair	Every year	112.66
	Drainage system cleaning and repair	Every year	187.76
	Routine cleaning, coating & structural protection of overbridge	Every 4 years	115.36
	Underbridge inspection for piers’ bases, scouring and submerged components	Every 6 years	435.86
	Serving of bearing	Every 4 years	73.51
	Detailed inspection of structures (steel & concrete components—underbridges and overbridges)	Every 2 years	136.91

**Table 5:** The material list for the Minnamurra Railway Bridge.

Component	Item	Material
Track	Transoms	FFU composites
	Transom bolts	Steel
	UIC 60 rail	Steel
	Ballast	Crushed aggregate
Substructure	Pier	Concrete
	Abutment	Steel/Concrete
Superstructure	Box girder	Steel
	Bracing used for box girder	Steel
	Bearings	Neoprene & natural rubber
Conductor rail	Insulation components	Galvanized steel/rubber
	Conductor rail	High conductivity steel
	Anchor assemblies	Galvanized steel
	Splice joint assembly	Stainless steel
	Power and cable assemblies	Copper/metal
Fastening system	Rail pads	High density polyethylene/natural rubber
	Baseplates	Steel
	Clip insulator	High density polyethylene/natural rubber
	Anchor assemblies	Galvanized steel
	Zero toe-load clips	Spring steel
	Normal fastening clips	Spring steel

**Table 6:** List of Maintenance Replacement and Repair Activities.

Causes	Actions
Small cracks, spalling occurs in concrete pier or concrete transoms	- determine the contaminants that cause cracking, such as chlorides, carbon dioxide sulphates, etc. - use Siloxane pore liner, Epoxy resin coating and Acrylic resin coating to prevent damage from deterioration of erosion against hostile environments
Track damaged by derailment	- check all track and structure components and verify with other departments/disciplines that the entire track segment has been inspected and repaired before returning track to service - check track stability and rail stress management and verify that rail stresses and creep marks have been adjusted appropriately to climate conditions - assess and commission all track conditions (including components/structures, track geometry, track alignment, and transit space) in accordance with base operating conditions (BOCs) to assure track integrity for safe operations
Bolted joints and welds in continuous welded rail (CWR) experiencing service failure	- check conditions and repair/replace the welds & joint components (e.g., expansion joints at bridge ends, mechanical joints, etc.) - the failure parts should be replaced/repaired immediately (e.g., bolds, bars, expansion joints, weld materials, etc.) - assess the weld and joint structure for potentially a full replacement appropriately by either welded joints or like-for-like bolted joints - assess weld damage and full rail profile (e.g., loss of materials, wear, dipped joint, spark erosion, plastic flow, chipped or broken weld, rail foot damage, hardness and heat-affected zone, localised misalignment, kink/bend, etc.) and repair/replace accordingly.
Rail seat abrasion due to deteriorated or incorrect pad and fastening assembly	- check if the abrasion rate whether it is corresponding to the standard (base operating conditions or BOCs). If not complied, the rail and fastening system (i.e., pads, insulators, clips, normal and zero-toe-load fasteners) should be replaced/repaired.

**Table 7:** Potential Failure Criteria for Bridges in Flood Events, modified from [78].

Element		Failure Criteria	Influence on Failure
Superstructure	Beam or girder	Unseating or loss of span	Collapse
	Deck	Damage due to debris and build-up of mud, undermining	Local damage, may contribute to collapse
	Approaches	Missing, damaged or obscured signs and delineation, guardrails	Does not lead to failure
		Blocked inlets/outlets	Some restrictions
		Missing, damaged, settlement or depression of track surface	Local damage, may lead to collapse, or may restrict the services
	Surface	Missing, damaged, scuppers blocked	Restrict use/service
Substructure	Pier or column	Movement, rotation and scour Momentary damage, shear damage, bending and shear damage, inadequate ductility capacity, poor durability	Local damage, may lead to collapse
	Abutment	Wingwall, backwall damage, inclination of abutment, damage to shear keys	Local damage, may lead to collapse
	Bearing	Missing, damaged or dislodged and poorly sealed	Local damage, may lead to collapse

## Data Availability

Data can be made available upon reasonable request to the corresponding author.
